# Supplementary material for: Systematic allelic analysis defines the interplay of key pathways in X chromosome inactivation
Source: Nat Commun. 2019 Jul 16;10:3129. doi: 10.1038/s41467-019-11171-3 (PMC6635394; doi:10.1038/s41467-019-11171-3)
Supplement: Supplementary file 1 — Supplementary Information [file 41467_2019_11171_MOESM1_ESM.pdf]

Supplementary Materials:

Systematic Allelic Analysis Defines the Interplay of Key Pathways in X Chromosome Inactivation

Nesterova *et al.* 2019

Developmental Epigenetics, Department of Biochemistry, University of Oxford

Contents:

Supplementary Figures 1-8

Supplementary Tables 1-4

Supplementary references

Supplementary Figure 1

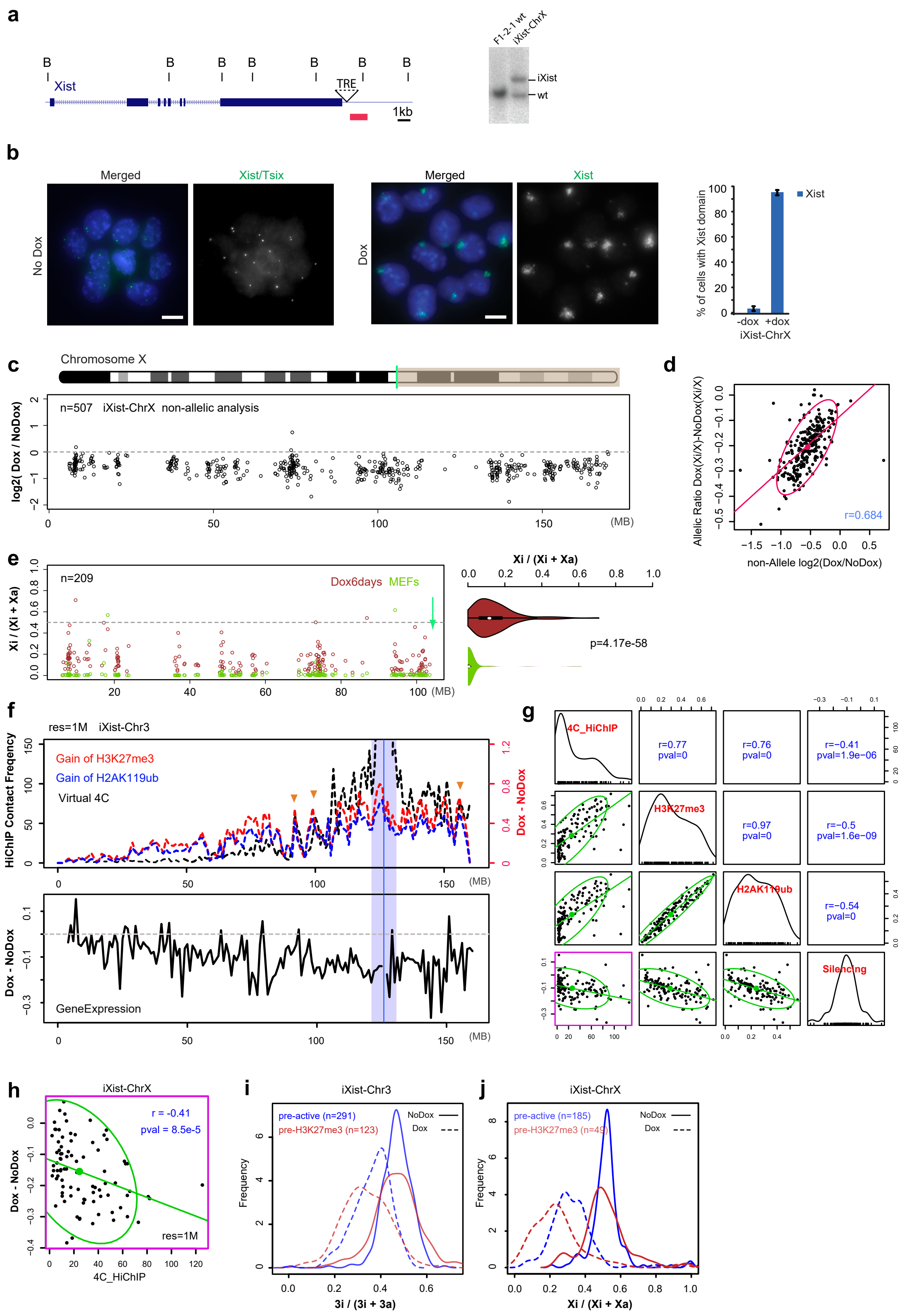

Supplementary Fig. 1. Supporting data for Fig. 1. **a**, Schematic illustrating exon/intron structure of Xist locus and insertion of TRE upstream of Xist exon 1 generated by CRISPR/Cas9-mediated homologous recombination. Integration site is indicated by a triangle; B, BamHI site; red bar is a Southern blot probe. Southern blot besides the schematic illustrates a changed size fragment of one of two Xist alleles, consistent with integration of TRE sequence in iXist cell line. Original Southern blot image is available from the Mendeley Data depository from this link [http://dx.doi.org/10.17632/52pjcxy486.1]. **b**, Example of RNA FISH analysis of Xist RNA (green) in iXist-ChrX cells without doxycycline induction (two left panels, Xist/Tsix) and after 1 day of doxycycline induction (two right panels). DNA is counterstained with DAPI (blue). Scale bar is 10μm. Bar chart on the right shows proportion of cells with Xist RNA domains in control (-dox) and after 1 day of doxycycline induction (+dox) based on scoring >200 cells in four independent experiments. Error bar indicates standard deviation. **c**, Gene silencing in iXist-ChrX cells after 1 day of Xist RNA induction as determined by non-allelic analysis. Grey shading on ChrX ideogram highlights distal region where informative SNPs are not present. Mean value of allelic ratio for each gene was calculated from biological replicates as detailed in Methods. Green line on ChrX ideogram indicates location of the Xist locus**.** **d**, Correlation analysis comparing genes proximal to Xist in iXist-ChrX cells analysed either by allelic or non-allelic methodology. Red oval indicates 95% of gene population. **e**, Comparison of silencing in iXist-ChrX cells after 6 days of Xist RNA induction with published dataset showing silencing in Cast x FVB XX somatic (MEF) line[1], shown for genes proximal to Xist locus. Mean value of difference of allelic ratio for each gene with an informative SNP was calculated from biological replicates as detailed in Methods. Data are summarised in the violin plot to the right. p-values are calculated using one-sided Wilcoxon rank sum test. **f**, Representation of iXist-Chr3 Xist transgene locus (blue line) 4C-HiChIP contact frequency (black dashed line) together with allelic H3K27me3 and H2AK119ub gain following 1 day of Xist RNA induction. Red arrowheads indicate selected highly correlated regions. Allelic silencing is plotted below. **g**, Correlation analysis showing relationship between 4C-HiChIP, gain of H3K27me3 and H2AK119ub, and allelic silencing in iXist-Chr3 cells after 1 day of Xist RNA induction. Correlation for 4C-HiChIP with silencing (r=-0.41) is highlighted (red box). **h**, Correlation between allelic silencing and proximity to Xist locus (determined from published 4C-HiChIP data) in iXist-ChrX cells after 1 day of Xist RNA induction. Green oval indicates 95% of gene population. **i, j**, Allelic repression with/without 1 day of Xist RNA induction determined for Chr3 (i) and ChrX (j) genes were grouped as having pre-existing active (pre-active) or H3K27me3 (pre-H3K27me3) chromatin signature.

Supplementary Figure 2

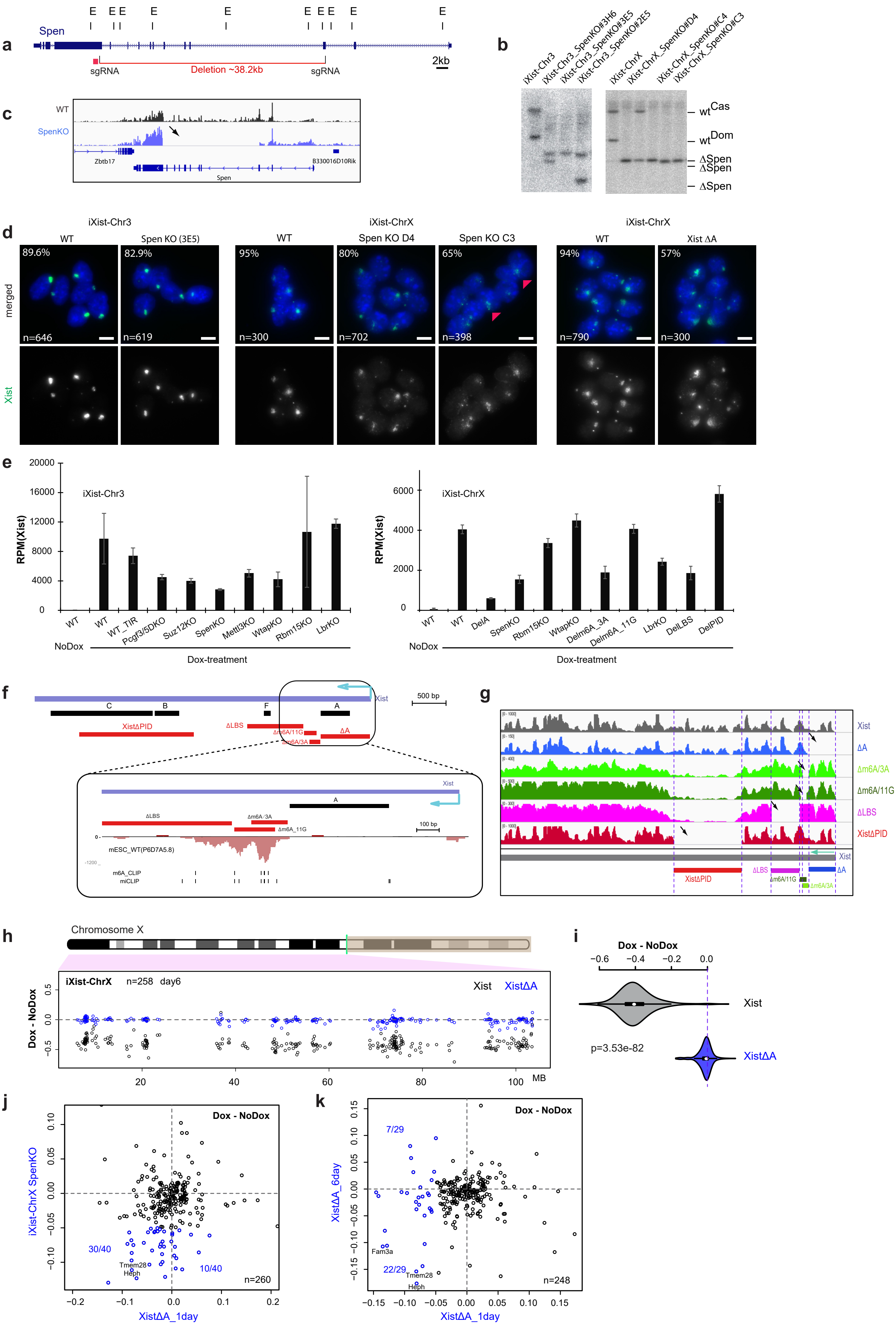

Supplementary Fig. 2. Supporting data for Fig. 2. a, Schematic illustrating exon/intron structure of *Spen* gene and position of the deletion generated by CRISPR/Cas9 mutagenesis. Location of sgRNAs is shown below the gene schematic. E, EcoRV site; red bar indicates the position of the Southern blot probe. **b**, Southern blot illustrating disappearance of wild type fragments in Spen mutant cell lines. Note that domesticus and castaneus strains have different fragment mobility due to sequence polymorphism. Original Southern blot image is available from the Mendeley Data depository from this link [http://dx.doi.org/10.17632/52pjcxy486.1]. **c**, UCSC screenshot illustrating lack of sequencing reads for the deleted region obtained from chromatin RNA sequencing of the cell lines. **d**, Representative images of RNA FISH analysis showing Xist RNA domains (green) in iXist-Chr3, iXist ChrX and derivative mutant cell lines as indicated. Percentage of cells with Xist domains and the number of cells scored for each cell line is shown. DNA is counterstained with DAPI (blue). Scale bar is 10μm. Arrowheads on Spen KO image indicate cells with diffuse Xist RNA domain. **e**, Xist RNA levels after 1 day of induction determined from ChrRNA-seq experiments and expressed as RPM(Xist) for iXist-Chr3 (left) and iXist-ChrX (right) and derivative mutant mESC lines. Error bars indicate s.e.m. **f**, Schematic showing the proximal region of the *Xist* gene indicating the location of different deletions generated in iXist-ChrX cells. The Xist promoter (cyan arrow) is shown for reference. Below zoomed-in region is m6A-seq data indicating the prominent m6A region in Xist RNA. Individual m6A sites determined by m6A-CLIP[2] and miCLIP[3] are also denoted. **g**, UCSC screenshot illustrating lack of sequencing reads in expected regions for the different Xist deletions generated by CRISPR/Cas9 mutagenesis. Data is obtained from chromatin RNA sequencing of the cell lines. Regions deleted in specific mutations are shown underneath and colour-coded the same as corresponding sequencing tracks. Arrows indicate the deletion regions. Reduced number of reads in XistPID region is due to low mappability of the reads over the B/C repetitive sequence. **h**, Allelic silencing across ChrX after 6 days of Xist RNA induction in WT and XistΔA mESCs. Mean value of difference of allelic ratio for each gene with an informative SNP was calculated from biological replicates as detailed in Methods. Green line on ChrX ideogram indicates location of the *Xist* locus. **i**, Violin plot summarising data in **(h)**. p-values were calculated using one-sided Wilcoxon rank sum test. **j**, Comparison showing silencing level for genes that are repressed in Spen KO (blue) after 1 day of Xist RNA induction in XistΔA mESCs. **k**, Comparison showing silencing level for genes that are repressed in XistΔA (blue) after 1 day and 6 days of Xist RNA induction in XistΔA mESCs.

Supplementary Figure 3

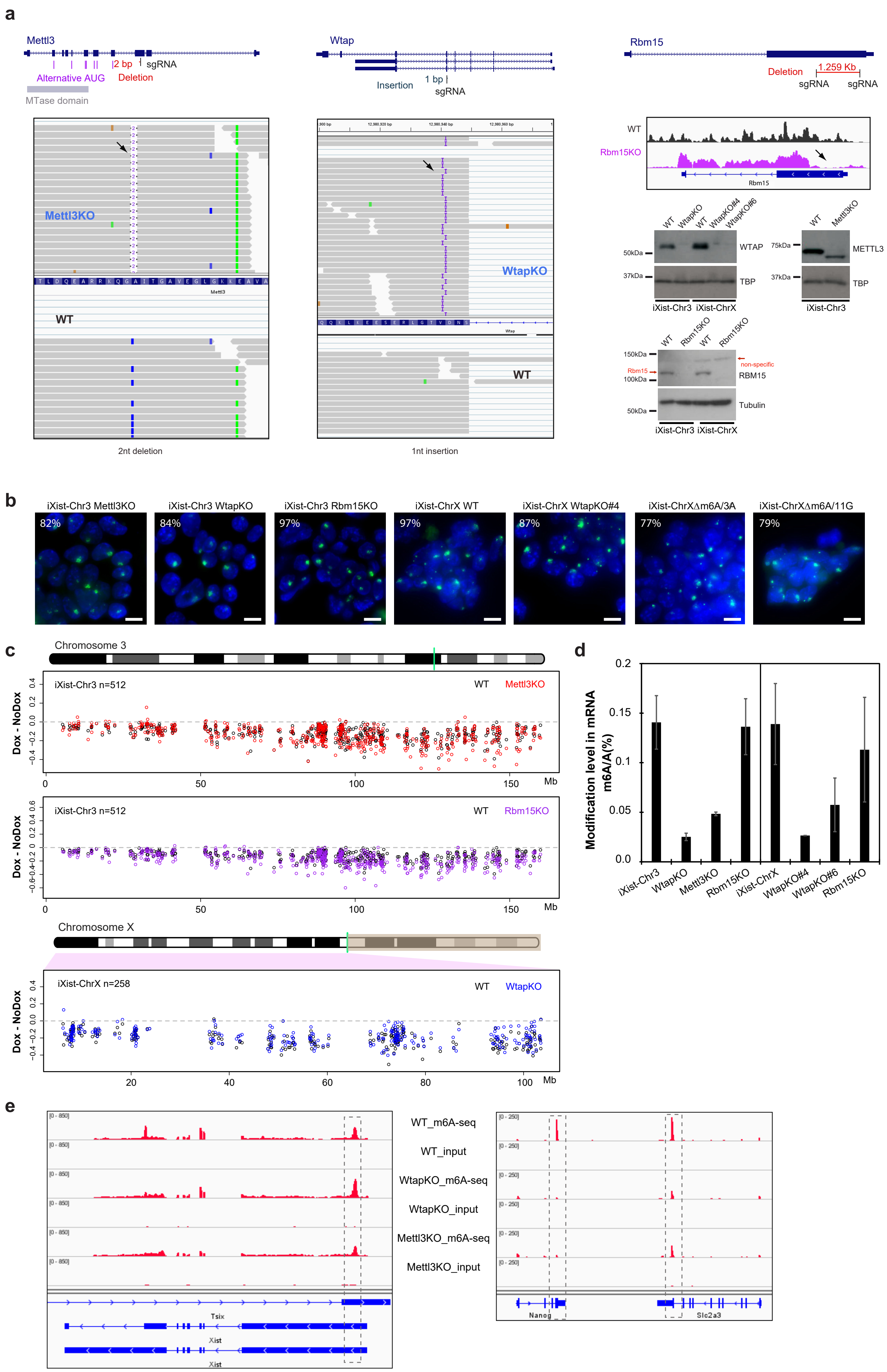

Supplementary Fig. 3. Supporting data for Fig. 3. a, Schematics illustrating mutations in *Mettl3*, *Wtap* and *Rbm15* genes generated by CRISPR/Cas9 mutagenesis. Black vertical bars indicate location of sgRNA complementary sequence. Vertical bars indicate alternative AUG in Mettl3 gene potentially giving rise to truncated catalytically active protein product. IGV browser screenshots illustrating chromatin RNA sequencing data across the *Mettl3* and *Wtap* genes are shown below corresponding gene schematic. UCSC screenshot illustrating almost complete lack of sequencing reads over the Rbm15 deletion is shown underneath Rbm15 schematic. Data is obtained from chromatin RNA sequencing of the cell lines. Western blot analysis of the Mettl3, Wtap and Rbm15 in corresponding mutant cell lines compared to the parental cell lines, iXist-Chr3 or iXist-ChrX. Lamin B and a-tubulin western blots are included as loading controls. Molecular weight markers are shown on the left. **b**, Representative RNA FISH images showing Xist RNA domains (green) in different iXist-Chr3 and iXist-ChrX mutant lines as indicated. Percentage of cells with Xist domains for each cell line is shown. At least 100 cells was scored for each line. DNA is counterstained with DAPI (blue). Scale bar is 10 μm. **c,** Allelic silencing across Chr3 after 1 day of Xist RNA induction in WT compared to Mettl3 mutant (KO) (top), in WT compared to Rbm15 null (KO) iXist-Chr3 mESCs (middle), and for WtapKO across ChrX in iXist-ChrX cells (bottom). Mean value of difference of allelic ratio for each gene with an informative SNP was calculated from biological replicates as detailed in Methods. Green lines on Chr3 and ChrX ideograms indicate location of the Xist transgene/locus. **d,** Levels of m6A in mRNA from iXist-Chr3 and iXist-ChrX mESCs and indicated mutant cell lines determined by LC-MS/MS. Two independent iXist-ChrX WtapKO cell lines were analysed. Error bars indicate variation between two biological replicates. **e,** IGV browser screenshots illustrating m6A-seq data across the *Xist* locus (left) and *Nanog* and *Slc2a3* loci (right) in WT iXist-Chr3 mESCs and derivative *Wtap* KO and *Mettl3* KO as indicated. Dotted boxes indicate major m6A peaks.

Supplementary Figure 4

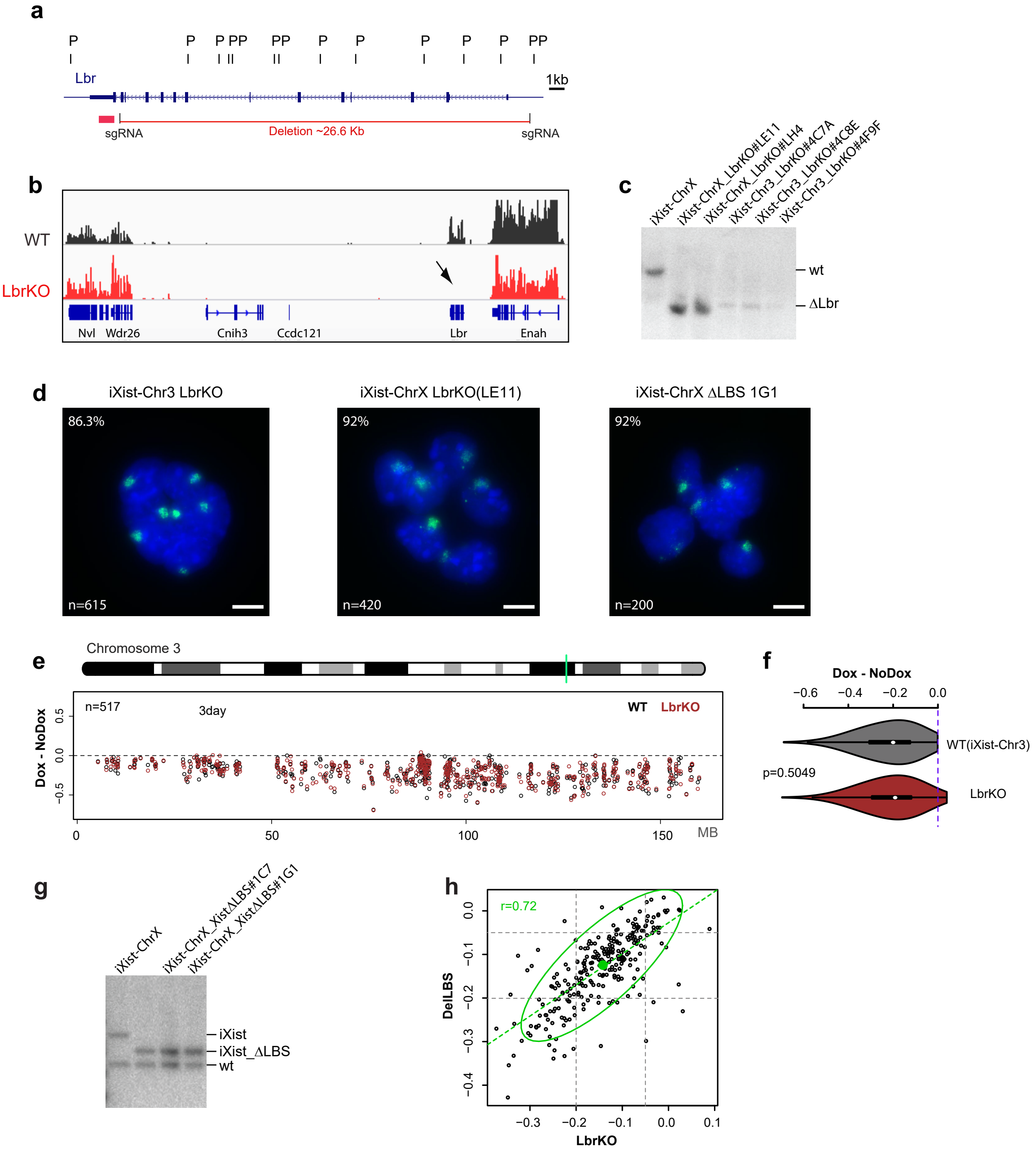

Supplementary Fig. 4. Supporting data for Fig. 4. a, Locus map of the *Lbr* gene showing the location of the deletion generated by CRISPR/Cas9 mutagenesis. P, PvuII site; red bar indicates the position of the Southern blot probe. **b**, UCSC screenshot illustrating lack of sequencing reads for the Lbr gene obtained from chromatin RNA sequencing of the cell lines. **c**, Southern blot illustrating disappearance of wild type fragment in Lbr mutant cell lines. **d**, Representative RNA FISH images showing Xist RNA domains (green) in different iXist-Chr3 and iXist-ChrX mutant lines as indicated. Percentage of cells with Xist domains and the number of cells scored for each cell line is shown. DNA is counterstained with DAPI (blue). Scale bar is 10 μm. **e,** Allelic silencing across Chr3 after 3 days of Xist RNA induction in WT compared to *Lbr* null (KO) iXist-Chr3 mESCs. Mean value of difference of allelic ratio for each gene with an informative SNP was calculated from biological replicates as detailed in Methods. Green line on Chr3 ideogram indicates location of the Xist transgene. **f,** Violin plot summarising data from **(e)**. p-values were calculated using one-sided Wilcoxon rank sum test. **g,** Southern blot illustrates a reduced size fragment of the Xist alleles carrying TRE promoter, consistent with deletion of the LBS element in iXist-ChrX_XistΔLBS cell lines. See schematic in Supplementary Fig.1a for the position of the restriction sites and Southern blot probe. All original Southern blot images are available from the Mendeley Data depository from this link [http://dx.doi.org/10.17632/52pjcxy486.1]. **h,** Correlation of gene silencing in *Lbr* null and XistΔLBS mESCs. Green oval represents 95% of the gene population.

Supplementary Figure 5

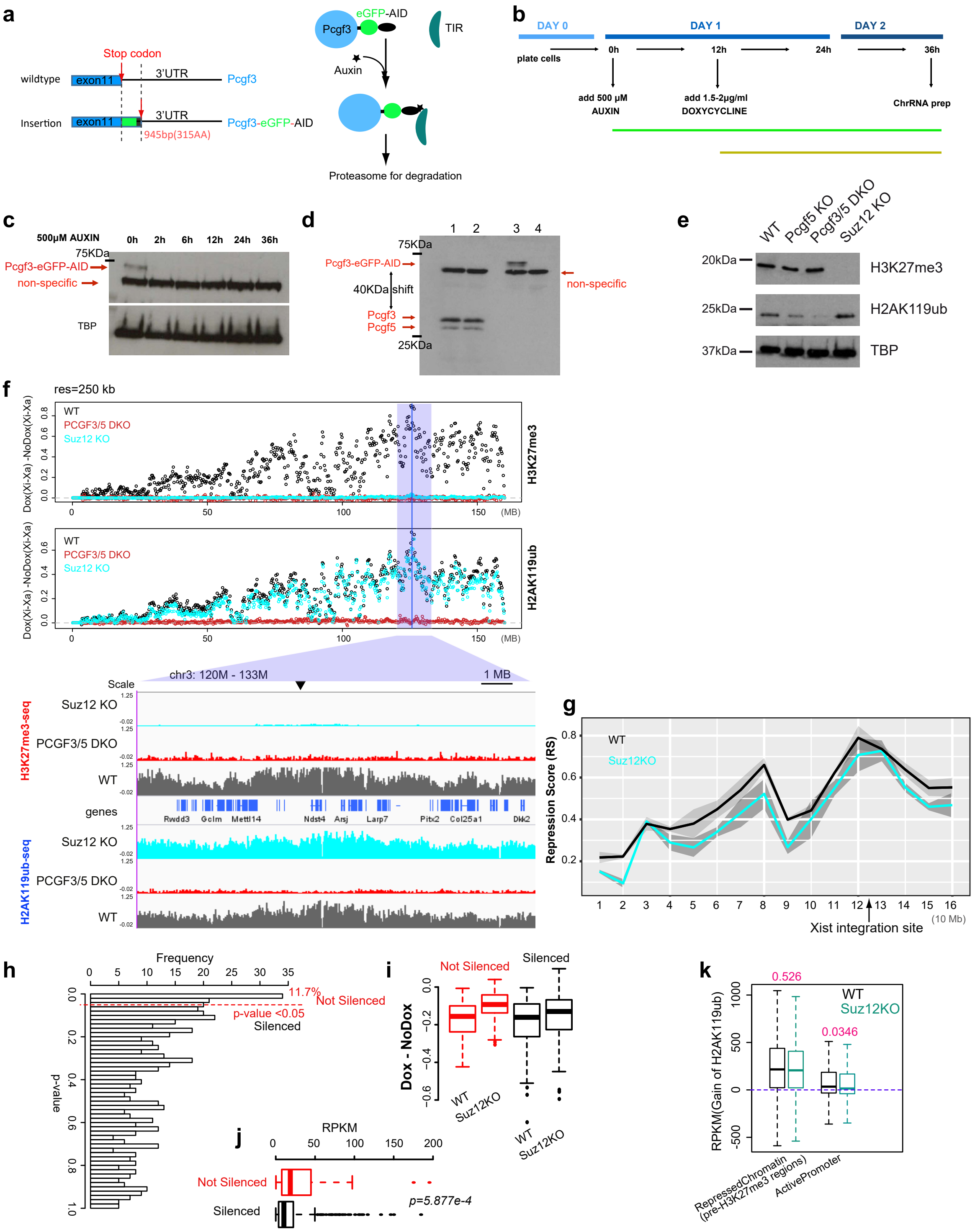

Supplementary Fig. 5. Supporting data for Fig. 5. **a,** Strategy for tagging *Pcgf3* exon 11 with an in frame GFP-AID tag. **b**, Schematic for PCGF3 degradation showing Auxin treatment (green line) and Xist RNA induction (yellow line) time windows. **c,** Western blot illustrating that degradation of PCGF3 in *Pcgf3/5* DKO mESCs occurs within 2 hours of addition of Auxin. TBP is a loading control. **d,** PCGF3 + PCGF5 western blot in WT iXist-Chr3 mESCs (lanes 1 and 2), and in *Pcgf3/5* DKO mESCs (lanes 3 and 4). Auxin was added for 36h (lanes 2 and 4). **e,** Western blot showing global H3K27me3 and H2AK119ub levels in cell lines as indicated. DKO is post-Auxin treatment. TBP is a loading control. **f,** Gain of H3K27me3 (top) and H2AK119ub (bottom) on Chr3 determined by calibrated allelic ChIP-seq after 1 day of Xist RNA induction in WT, *Pcgf3/5* DKO and *Suz12* KO iXist-Chr3 mESCs as indicated. The location of the Xist transgene is shown with a blue vertical line. IGV browser screenshot zoomed to 13Mb around the Xist transgene is shown below. **g,** Allelic silencing illustrated as repression score[4], for WT and *Suz12* KO mESCs after 3 day of Xist RNA induction. **h,** Frequency of genes that are not silenced ($p<0.05$) in *Suz12* KO compared to WT mESCs after 3 days of Xist RNA induction. **i,** Boxplot illustrating allelic silencing in *Suz12* KO for genes classified in (H) as Not silenced and Silenced. **j,** Boxplot illustrating Not Silenced and Silenced genes in *Suz12* KO plotted against expression level in mESCs. p-value is calculated from two-sided Wilcoxon rank sum test. **k,** Boxplot illustrating gain of H2AK119ub at genes with pre-existing H3K27me3 (pre-H3K27me3) or active (pre-active) chromatin marks in WT and *Suz12* KO mESCs after 1 day of Xist RNA induction. p-value is calculated from two-sided Wilcoxon rank sum test. For all the boxplots (i-k) the lower and upper edge of the box represent the first and third quartile, respectively. The horizontal line inside the box indicates the median. Whiskers identify the farthest data points within 1.5× the interquartile range (IQR).

Supplementary Figure 6

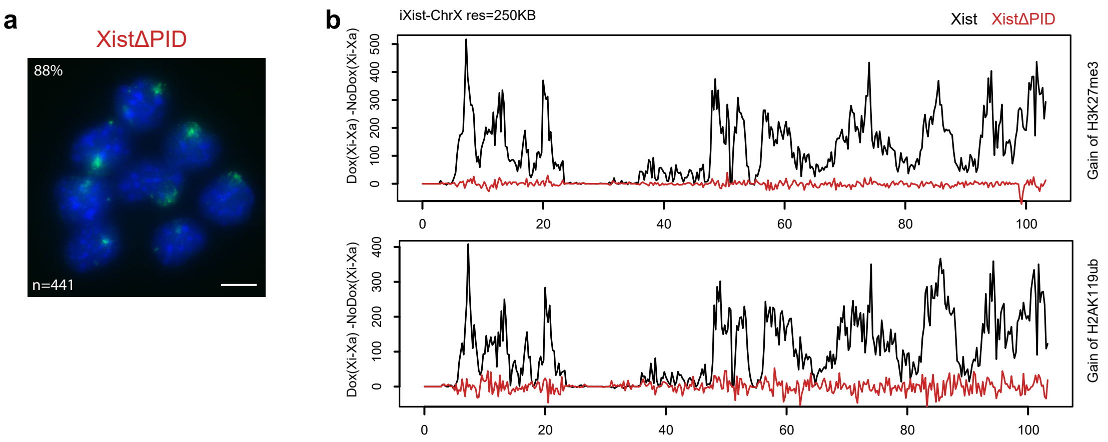

Supplementary Fig. 6. Supporting data for Figs. 5 and 6. **a,** Representative image of RNA FISH analysis showing Xist RNA domains (green) in iXist-ChrX XistΔPID mESCs. Percentage of cells with Xist domains and the number of cells scored is shown. DNA is counterstained with DAPI (blue). Scale bar is 10μm **b,** Gain of H3K27me3 (top) and H2AK119ub (bottom) in 500kb bins on Xist proximal ChrX determined by calibrated allelic ChIP-seq after 1 day of Xist RNA induction in iXist-ChrX and XistΔPID mESCs.

Supplementary Figure 7

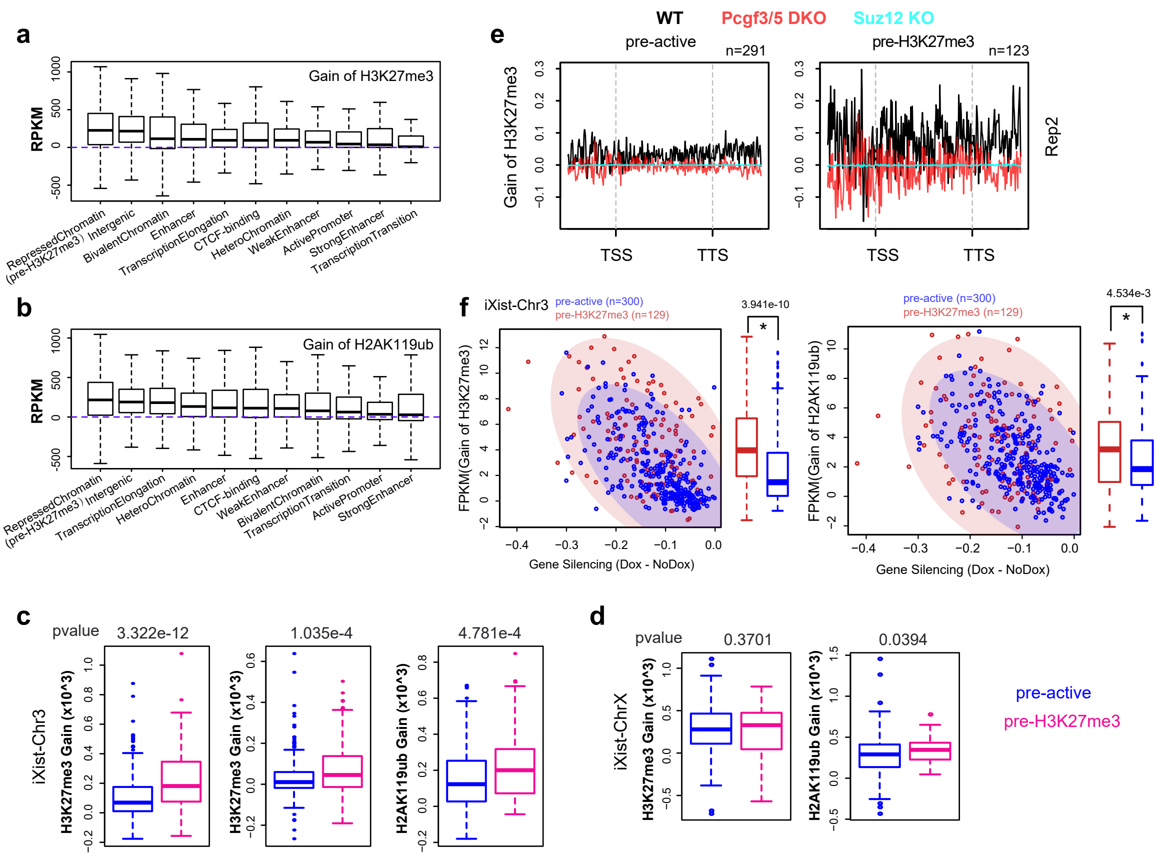

Supplementary Fig. 7. Supporting data for Fig. 6. a, b, ChromHMM analysis showing gain of H3K27me3 **(a)** and H2AK119ub **(b)** at different regions defined by epigenetic chromatin state in iXist-Chr3 mESCs after 1 day of Xist RNA induction. **c, d,** Statistical analysis of Polycomb mark (H3K27me3 and H2AK119ub) gain in iXist-Chr3 **(c)** and iXist-ChrX **(d)** respectively for genes with pre-active (blue) and pre-H3K27me3 (pink) signatures. The significance level was determined by one-side Wilcoxon rank-sum test in R. **e,** Metaprofile complementing Fig. 6g, h using a different biological replicate. **f,** Scatterplots and boxplots illustrating increased gain of H3K27me3 (left) and H2AK119ub (right) in gene promoters with pre-existing H3K27me3 (pre-H3K27me3) compared with active (pre-active) chromatin marks in iXist-Chr3 mESCs after 1 day of Xist RNA induction. For all the boxplots the lower and upper edge of the box represent the first and third quartile, respectively. The horizontal line inside the box indicates the median. Whiskers identify the farthest data points within 1.5× the interquartile range (IQR).

Supplementary Figure 8

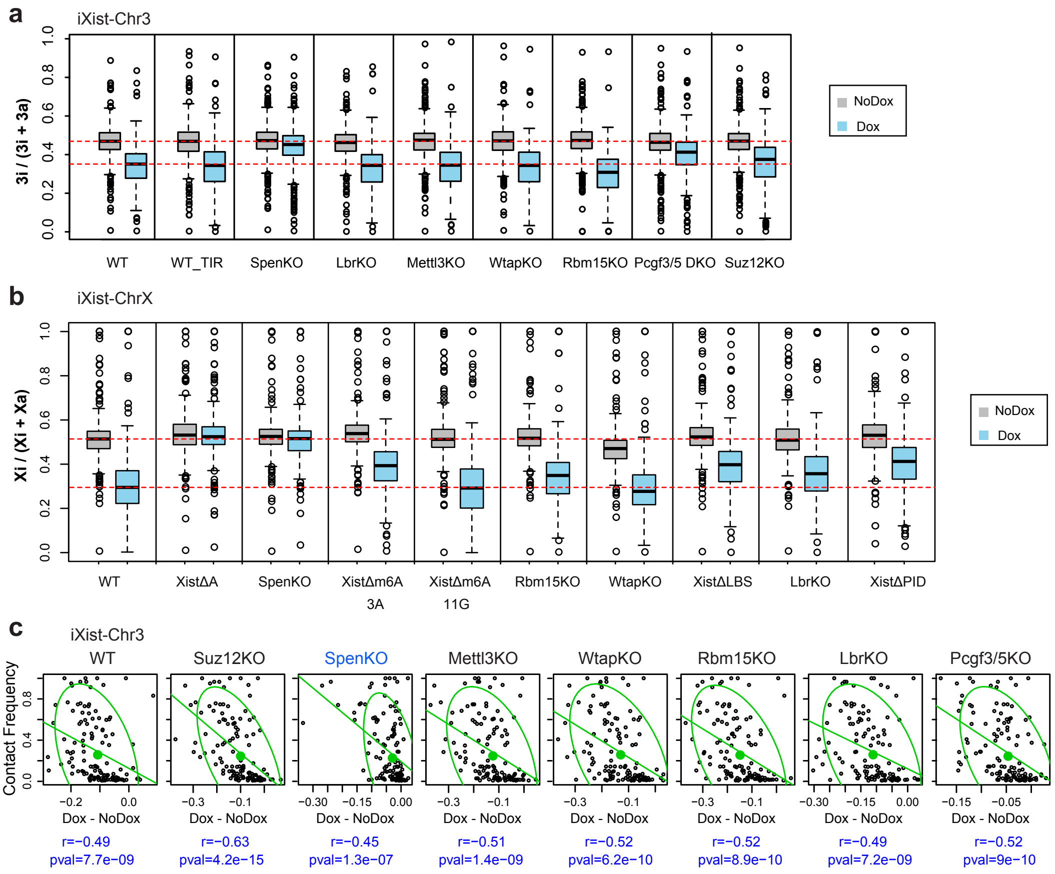

Supplementary Fig. 8. Supporting data for Fig. 7. a, Boxplots summarising allelic silencing after 1 day of Xist RNA induction in iXist-Chr3 and different mutant derivative mESCs. WT values represent an average of all determinations except for Pcgf3/5 DKO where the WT-TIR cell line is the appropriate control. **b**, As in (a) for iXist-ChrX. For all the boxplots the lower and upper edge of the box represent the first and third quartile, respectively. The horizontal line inside the box indicates the median. Whiskers identify the farthest data points within 1.5× the interquartile range (IQR). **c**, Correlation analysis for topological distance and silencing efficiency for WT and selected knockout mESCs in iXist-Chr3 model. 95% of the population is encircled. Spearman correlation and p-values are indicated below.

Supplementary Table 1. Cell Lines used in this study and validation methods used to characterized each line

mESC: iXist-Chr3, XY	WB, RNA-FISH, RNAseq
mESC: iXist-Chr3 LBR KO, XY	gPCR, SS, SB, RNAseq
mESC: iXist-Chr3 METTL3 KO, XY	gPCR, SS, WB, RNA-FISH, RNAseq
mESC: iXist-Chr3 PCGF3-AID PCGF5 KO, XY	gPCR, SS, WB
mESC: iXist-Chr3 RBM15 KO, XY	SS, WB, RNAseq
mESC: iXist-Chr3 SPEN KO, XY	gPCR, SS, SB, RNAseq
mESC: iXist-Chr3 WTAP KO, XY	gPCR, SS, WB, IF, RNA-FISH, RNAseq
mESC: iXist-ChrX, XX	gPCR, SS, SB, IF, RNA-FISH, mFISH, RNAseq, ChIPseq
mESC: iXist-ChrX LBR KO, XX	gPCR, SS, SB, RNAseq
mESC: iXist-ChrX RBM15 KO, XX	gPCR, WB, RNAseq
mESC: iXist-ChrX SPEN KO, XX	gPCR, SS, SB, RNAseq
mESC: iXist-ChrX WTAP KO B2.4, XX	gPCR, SS, IF, RNA-FISH, RNAseq
mESC: iXist-ChrX WTAP KO B2.6, XX	gPCR, SS, IF, RNA-FISH, RNAseq
mESC: iXist-ChrX Xist ΔA, XX	gPCR, SS, RNAseq
mESC: iXist-ChrX XistΔLBS KO, XX	gPCR, SS, SB, RNAseq
mESC: iXist-ChrX XistΔm6A 3A, XX	gPCR, RD, SS, RNA-FISH, RNAseq
mESC: iXist-ChrX Xist Δm6A 11G.7, XX	gPCR, RD, SS, RNA-FISH, RNAseq
mESC: iXist-ChrX Xist ΔPID, XX	gPCR, SS, RNAseq

Supplementary Table 2. List of Oligonucleotides

Oligonucleotide	Source	Used for
LBR_gRNA1, ATGATGTTGAATTCGTATAC	This study	CRISPR KO
LBR_gRNA2, GCCTACCTTATGAGCGGGTT	This study	CRISPR KO
LBR_F, GTTAAATGTTAAGGAAAAGCAGG	This study	gPCR, sequencing
LBR_R, TCACCCACTGAAAGCCAAGC	This study	gPCR, sequencing
METTL3_gRNA, ACGCCGTTTCTGCCCTGCGATGG	This study	CRISPR KO
METTL3_F, CAGCGTGCTTCCTTGACTTCTT	This study	gPCR, sequencing
METTL3_R, TCCAAGATGATGCACATCCTACTCT	This study	gPCR, sequencing
PCGF3_gRNA1, CCAGCACGCGTTTACAGAGGA	This study	CRISPR HR
PCGF3_F, GAAAGGTTGAACGTGCACCT	This study	gPCR, sequencing
PCGF3_R, GCGAGCTGTTTCCTCAAGTG	This study	gPCR, sequencing
PCGF5_gRNA1, GCTCCTCTGCTTCAACTGCT	This study	CRISPR KO
PCGF5_gRNA2, GATCAAGCCCACGACAGTGACGG	This study	CRISPR KO
PCGF5_F, TGTTTACAGAGAGGAAGCGCC	This study	gPCR, sequencing
PCGF5_R, TGGCCTTGGTACACATATAGC	This study	gPCR, sequencing
RBM15_gRNA1, TCACCGCCACGGGAGCGCTT	This study	CRISPR KO
RBM15_gRNA2, GCACGAGAATTTGACCGATT	This study	CRISPR KO
RBM15_F, GGAGTCCAAGATGGCGGCGTG	This study	gPCR, sequencing
RBM15_R, CACTAGTTCATAGTGGGTCAAGG	This study	gPCR, sequencing
SPEN_gRNA3, GGGGTGTCTCCTGCGCATT	Suppl.Ref.5	CRISPR KO
SPEN_gRNA5, CGGACAAGACATTACGATC	Suppl.Ref.5	CRISPR KO
SPEN_F, CAGAAAGAGGCGAGGCGTAAAG	This study	gPCR, sequencing
SPEN_R, GCGCTCCAGCCGAGCCTTCTC	This study	gPCR, sequencing
TIGRE_gRNA, ACTGCCATAACACCTAACTT	M.Houlard	CRISPR HR
TIGRE_rtTA_F, AAGGGGGAGGATTGGGAAGAC	This study	gPCR, sequencing
TIGRE_R, GTGATCCACGGTGATCCACA	M.Houlard	gPCR, sequencing
WTAP_gRNA, TACTCAGCAAACGATGTGACTGG	This study	CRISPR KO
WTAP_F, GTAGTCCCTTGATGCAAAGTA	This study	gPCR, sequencing
WTAP_R, AACAGTTTTGTTTTGAGGAG	This study	gPCR, sequencing
Xist_TRE_gRNA1, TAACTGATCCGCGGCGCTGA	This study	CRISPR HR
Xist_TRE_gRNA2, GCACGCCTTTAACTGATCCG	This study	CRISPR HR
Xist ΔA_gRNA, GATCAGTTAAAGGCGTGCAA	This study	CRISPR HR
Xist ΔA_F, TACCCGGGGATCCTCTAGTC	This study	gPCR, sequencing
Xist ΔA_R, CGGTGTCCTAATTCTTGGCG	This study	gPCR, sequencing
XistΔLBS_gRNA1, TTTAGCTAGCGCAGCGCAAT	This study	CRISPR HR
XistΔLBS_gRNA2, AAGCATGCGCTCTCCCGACC	This study	CRISPR HR on 129 allele
XistΔLBS_F, ACGGCTATTCTCGAGCCAGTT	This study	gPCR, sequencing
XistΔLBS_R, GGACTCCAAAGTAACAATTC	This study	gPCR, sequencing
XistΔm6A_gRNA1, TTTTTTCGAAGTGCCTGCCCAGG	This study	CRISPR HR on 129 allele
XistΔm6A_F, TTTTTTTTCACGGCCCAACGGGGCG	This study	gPCR, sequencing
XistΔm6A_R, ATACCGCACCAAGAACTTGAGCC	This study	gPCR, sequencing
Xist ΔPID_gRNA, TTGAGGTTCTATACCAGTTC	This study	CRISPR HR
Xist ΔPID_F, TCAAGCGGTTCTCTAAGCCT	This study	gPCR, sequencing
Xist ΔPID_R, : GATGATACCCTCCCATGGCA	This study	gPCR, sequencing

Supplementary Table 3. List of Antibodies used in the study

Antibody	Company	Cat. number
WTAP, Rabbit, WB, IF (1:1000)	Proteintech	Cat#10200-1-AP
METTL3, Rabbit, WB, IF (1:1000)	Abcam	Cat#ab195352
m6A, Rabbit, M6A –seq (1μg/30μg RNA)	Synaptic systems	Cat#202003
H2AK119ub, Rabbit, ChIP (4μl/5x10^6cells)	Cell Signaling	Cat#8240S
H3K27me3, Rabbit, ChIP (4μl/5x10^6cells)	Diagenode	Cat# C15410069
H3K27me3, Mouse, IF (1:1000)	Active Motif	Cat# 61017
RBM15, Rabbit, WB (1:2000)	Proteintech	Cat# 10587-1-AP
PCGF3+PCGF5, WB (1:1000)	Abcam	Cat#ab201510
TBP, Mouse, WB (1:3000)	Abcam	Cat#ab51841
Tubulin, Rabbit, WB (1:1000)	Cell Signaling	Cat#2144
Alexa 568 anti-mouse IgG, Goat, IF (1:500)	Life Technologies	cat#A11031
Alexa 488 anti-mouse IgG , IF (1:500)	Life Technologies	cat#A11029
Alexa 568 anti-rabbit IgG, Goat, IF (1:500)	Life Technologies	cat#A11034
Alexa 488 anti-rabbit IgG, Goat, IF (1:500)	Life Technologies	cat#A11008
Anti-rabbit Ig, HPR, Donkey, WB (1:2000)	Amersham	cat#NA934V
Anti-mouse IgG, HRP, Sheep, WB (1:2000)	Amersham	cat#NXA931

Supplementary Table 4. Biological Replicate RNA Sequencing

Cell lines	ES/DIF, treatment time	+Dox Replicates	No Dox Replicates	Sequencing
iXist-Chr3	ES, 1 day	3	4	ChrRNA
iXist-Chr3_Pcgf5KO_TIR	ES, 1 day	2	1	ChrRNA
iXist-Chr3_Pcgf5KO_TIR_B7	ES, 1 day	2	1	ChrRNA
iXist-Chr3_Pcgf5KO_TIR_C10	ES, 1 day	2	1	ChrRNA
iXist-Chr3_Suz12KO	ES, 1 day	2	1	ChrRNA
iXist-Chr3_SpenKO#2E5	ES, 1 day	1	1	ChrRNA
iXist-Chr3_SpenKO#2G9	ES, 1 day	1	1	ChrRNA
iXist-Chr3_SpenKO#3E5	ES, 1 day	1	1	ChrRNA
iXist-Chr3_SpenKO#3H6	ES, 1 day	1	1	ChrRNA
iXist-Chr3_LbrKO#4C7A	ES, 1 day	1	1	ChrRNA
iXist-Chr3_LbrKO#4C8E	ES, 1 day	1	1	ChrRNA
iXist-Chr3_LbrKO#4F9F	ES, 1 day	1	1	ChrRNA
iXist-Chr3_Mettl3KO	ES, 1 day	2	1	ChrRNA
iXist-Chr3_WtapKO	ES, 1 day	2	1	ChrRNA
iXist-Chr3_Rbm15KO	ES, 1 day	2	1	ChrRNA
iXist-Chr3	DIF, 3 days	3	3	ChrRNA
iXist-Chr3_Suz12KO	DIF, 3 days	3	3	ChrRNA
iXist-Chr3_LbrKO#4C7A	DIF, 3 days	1	1	ChrRNA
iXist-Chr3_LbrKO#4C8E	DIF, 3 days	1	1	ChrRNA
iXist-Chr3_LbrKO#4F9F	DIF, 3 days	1	1	ChrRNA
iXist-ChrX	ES, 1 day	6	5	ChrRNA
iXist-ChrX_XistΔA	ES, 1 day	2	1	ChrRNA
iXist-ChrX_XistΔPID	ES, 1 day	2	1	ChrRNA
iXist-ChrX_XistΔLBS#1G1	ES, 1 day	1	1	ChrRNA
iXist-ChrX_XistΔLBS#1C7	ES, 1 day	1	1	ChrRNA
iXist-ChrX_LbrKO#LE11	ES, 1 day	1	1	ChrRNA
iXist-ChrX_LbrKO#LH4	ES, 1 day	1	1	ChrRNA
iXist-ChrX_Rbm15KO#E10	ES, 1 day	3	3	ChrRNA
iXist-ChrX_Rbm15KO#A8	ES, 1 day	4	3	ChrRNA
iXist-ChX_SpenKO#C3	ES, 1 day	1	1	ChrRNA
iXist-ChX_SpenKO#C4	ES, 1 day	1	1	ChrRNA
iXist-ChX_SpenKO#D4	ES, 1 day	1	1	ChrRNA
iXist-ChrX_Δm6A/3A	ES, 1 day	2	2	ChrRNA
iXist-ChrX_Δm6A/11G	ES, 1 day	2	2	ChrRNA
iXist-ChrX_WtapKO#4	ES, 1 day	2	1	ChrRNA
iXist-ChrX_WtapKO#6	ES, 1 day	2	1	ChrRNA
iXist-ChrX	ES, 6 days	1	1	ChrRNA
iXist-ChrX_XistΔA	ES, 6 days	1	1	ChrRNA
iXist-ChrX_XistΔPID	ES, 6 days	1	1	ChrRNA
iXist-ChrX	DIF, 6 days	3	3	ChrRNA
iXist-ChrX_XistΔA	DIF, 6 days	3	3	ChrRNA
iXist-ChrX_XistΔPID	DIF, 6 days	3	3	ChrRNA

Supplementary References

1. Gdula, M. R. *et al.* The non-canonical SMC protein SmcHD1 antagonises TAD formation and compartmentalisation on the inactive X chromosome. *Nature communications* **10**, 30, doi:10.1038/s41467-018-07907-2 (2019).

2. Ke, S et al. A majority of m6A residues are in the last exons, allowing the potential for 3' UTR regulation. *Genes Dev*. **29**, 2037-53, doi: 10.1101/gad.269415.115 (2015).

3. Linder, B. *et al.* Single-nucleotide-resolution mapping of m6A and m6Am throughout the transcriptome. *Nature methods* **12**, 767-772, doi:10.1038/nmeth.3453 (2015).

4. Pintacuda, G. *et al.* hnRNPK Recruits PCGF3/5-PRC1 to the Xist RNA B-Repeat to Establish Polycomb-Mediated Chromosomal Silencing. *Molecular Cell* **68**, 955-969 e910, doi:10.1016/j.molcel.2017.11.013 (2017).

5. Monfort, A. *et al.* Identification of Spen as a Crucial Factor for Xist Function through Forward Genetic Screening in Haploid Embryonic Stem Cells. *Cell reports* **12**, 554-561, doi:10.1016/j.celrep.2015.06.067 (2015).
