# Supplementary material for: Systematic allelic analysis defines the interplay of key pathways in X chromosome inactivation
Source: Nat Commun. 2019 Jul 16;10:3129. doi: 10.1038/s41467-019-11171-3 (PMC6635394; doi:10.1038/s41467-019-11171-3)
Supplement: Supplementary file 3 — Reporting Summary [file 41467_2019_11171_MOESM3_ESM.pdf]

nature research

Corresponding author(s): Neil Brockdorff

# Reporting Summary

Nature Research wishes to improve the reproducibility of the work that we publish. This form provides structure for consistency and transparency in reporting. For further information on Nature Research policies, see Authors & Referees and the Editorial Policy Checklist.

## Statistical parameters

When statistical analyses are reported, confirm that the following items are present in the relevant location (e.g. figure legend, table legend, main text, or Methods section).

| n/a | Confirmed | |
|---|---|---|
| ☐ | ☒ | The exact sample size (*n*) for each experimental group/condition, given as a discrete number and unit of measurement |
| ☐ | ☒ | An indication of whether measurements were taken from distinct samples or whether the same sample was measured repeatedly |
| ☐ | ☒ | The statistical test(s) used AND whether they are one- or two-sided<br>*Only common tests should be described solely by name; describe more complex techniques in the Methods section.* |
| ☒ | ☐ | A description of all covariates tested |
| ☐ | ☒ | A description of any assumptions or corrections, such as tests of normality and adjustment for multiple comparisons |
| ☐ | ☒ | A full description of the statistics including central tendency (e.g. means) or other basic estimates (e.g. regression coefficient) AND variation (e.g. standard deviation) or associated estimates of uncertainty (e.g. confidence intervals) |
| ☐ | ☒ | For null hypothesis testing, the test statistic (e.g. *F*, *t*, *r*) with confidence intervals, effect sizes, degrees of freedom and *P* value noted<br>*Give P values as exact values whenever suitable.* |
| ☒ | ☐ | For Bayesian analysis, information on the choice of priors and Markov chain Monte Carlo settings |
| ☒ | ☐ | For hierarchical and complex designs, identification of the appropriate level for tests and full reporting of outcomes |
| ☐ | ☒ | Estimates of effect sizes (e.g. Cohen's *d*, Pearson's *r*), indicating how they were calculated |
| ☐ | ☒ | Clearly defined error bars<br>*State explicitly what error bars represent (e.g. SD, SE, CI)* |

*Our web collection on statistics for biologists may be useful.*

## Software and code

Policy information about availability of computer code

| | |
|---|---|
| Data collection | Data were collected using Illumina (NextSeq 500) next-generation sequencing platforms. Sequencing data were directly downloaded from BaseSpace. |
| Data analysis | RNA-seq data analysis<br>The following analysis strategy was used for Chromatin RNA-seq and mRNA-seq. Briefly, the raw fastq files of read pairs were first mapped to an rRNA build by bowtie2 (v2.3.2) and rRNA-mapped reads discarded. The remaining unmapped reads were aligned to the 'N-masked' genome (from mm10 coordinates) with STAR (v2.4.2a) using parameters "--outFilterMultimapNmax 1 --outFilterMismatchNmax 4 --alignEndsType EndToEnd" for all the sequencing libraries. Unique alignments were retained for further analysis. We made use of 23,005,850 SNPs between Cast and 129S genomes and employed SNPsplit (v0.2.0, Babraham Institute, Cambridge, UK) to split the alignment into distinct alleles (Cast and 129S) using the parameter "--paired". The (allelic) read numbers were counted by the program featureCounts (-t transcript -g gene_id -s 2) and the alignments were sorted by Samtools. Bigwig files were generated by Bedtools and visualized by IGV or UCSC Genome Browser. For biallelic analysis, counts were normalized to 1 million mapped read pairs (as CPM) by the edgeR R package. Genes with at least 10 SNP-covering reads across all the samples were further taken to calculate the allelic ratio of Xi/(Xi+Xa) where Xi and Xa indicate inactive and active allele, respectively.<br>In the iXist-Chr3 XY cell line the inducible Xist transgene expresses from the Cast allele, whereas in the iXist-ChrX XX line the endogenous Xist from the 129S allele is induced. For some purposes we also used data from ChrRNA-seq performed in a reciprocal cell line iXist-ChrX XX C7H8 in which endogenous Xist from the Cast allele is induced. Silencing is primarily quantified in all cell lines i.e. wild-type Xist, Xist mutants, and Xist-interacting factor KOs by the difference in allelic ratios between uninduced and induced samples, such that Gene Silencing (z)=[Xi/(Xi+Xa)]_Dox - [Xi/(Xi+Xa)]_NoDox |

For further comparisons, we set thresholds of z>-0.05, -0.05<z<-0.2, z<-0.2 to represent genes demonstrating weak/no, low, and high silencing respectively. Additionally, original levels of gene expression for each cell line were calculated as FPKM by Cuffnorm (v2.2.1), and used to further categorize genes into 3 graded expression groups (E1<E2<E3) with the same number of genes per group.
Data generated in Suz12 KO lines and corresponding wild-type iXist-Chr3 after 3 days in differentiation media was processed with the same pipeline as above. The p-values were calculated by Student's T-test based on the 3 biological replicates with 0.05 as the significance cutoff. PCA analysis was performed by R function prcomp and plotted with the R package ggplot2.

Native ChIP-seq calibration and data analysis
For ChIP-seq experiments quantitatively calibrated with Drosophila SG4 cells, raw fastq read pairs were mapped to the 'N-masked' mm10 genome concatenated with the dm6 genome sequence by STAR (v2.4.2a) using the same parameters as for RNA-seq data with the additional parameter "--alignIntronMax 10". Allelic split of mm10-mapped reads was conducted by SNPsplit as for RNA-seq. We then normalized the mm10-mapped reads to spiked-in Drosophila library size, and scaled down the WT sample to 10 million reads and the other calibrated samples by the same factor, for processing by Bedtools into bedGraph files for normalized signal. All the parameters and normalization intermediates can be found in Supplementary Table. We calculated absolute gain of H3K27me3 and H2AK119ub upon Xist induction by the formula (Xi-Xa)_Dox - (Xi-Xa)_NoDox for further analysis and visualization as bigWig files. Custom scripts (ExtractInfoFrombedGraph_AtBed.py) were used to extract values from sorted bedGraph files for different regions (e.g. 250 kb windows or ChromHMM annotations), with signal from regions of different sizes comparable by FPKM. Metagene profiles for pre-active and pre-H3K27me3 gene sets were generated by DANPOS with "--bin_size 50" and otherwise default parameters.

m6A-seq data analysis
For conventional m6A-seq data, first we removed the rRNA reads computationally by mapping the single-end reads to the mouse rRNA build with Bowtie2. The remaining unmapped reads were then aligned to mm10 genome by STAR (v2.4.2a) with "--single-end" mode. BigWig files were generated by Bedtools and normalized to 10 million mapped reads (Generate_BigWig_from_RNA_seq_Bam_mm10.sh).

For manuscripts utilizing custom algorithms or software that are central to the research but not yet described in published literature, software must be made available to editors/reviewers upon request. We strongly encourage code deposition in a community repository (e.g. GitHub). See the Nature Research guidelines for submitting code & software for further information.

## Data

Policy information about availability of data

All manuscripts must include a data availability statement. This statement should provide the following information, where applicable:

- Accession codes, unique identifiers, or web links for publicly available datasets
- A list of figures that have associated raw data
- A description of any restrictions on data availability

All raw and processed data are available in the GEO SuperSeries GSE119607

# Field-specific reporting

Please select the best fit for your research. If you are not sure, read the appropriate sections before making your selection.

☒ Life sciences ☐ Behavioural & social sciences ☐ Ecological, evolutionary & environmental sciences

For a reference copy of the document with all sections, see nature.com/authors/policies/ReportingSummary-flat.pdf

# Life sciences study design

All studies must disclose on these points even when the disclosure is negative.

| | |
|---|---|
| Sample size | In general, two to four independent biological replicates for each knockout were collected. For WT, which is used for comparison across all samples, we generated 5-6 replicates by different scientists in the lab |
| Data exclusions | No data exclusions |
| Replication | All experimental observations were successfully replicated and results reliably reproduced. The replicates number for each line can be found in Supplementary Table 4 |
| Randomization | not relevant |
| Blinding | All data were analyzed in an unbiased way using the exact same procedure irrespective of the cell type |

# Reporting for specific materials, systems and methods

## Materials & experimental systems

| n/a | Involved in the study | |
|---|---|---|
| ☒ | ☐ | Unique biological materials |
| ☐ | ☒ | Antibodies |
| ☐ | ☒ | Eukaryotic cell lines |
| ☒ | ☐ | Palaeontology |
| ☒ | ☐ | Animals and other organisms |
| ☒ | ☐ | Human research participants |

## Methods

| n/a | Involved in the study | |
|---|---|---|
| ☐ | ☒ | ChIP-seq |
| ☒ | ☐ | Flow cytometry |
| ☒ | ☐ | MRI-based neuroimaging |

# Antibodies

Antibodies used

WTAP, Rabbit, WB, IF Proteintech Cat#10200-1-AP
METTL3, Rabbit, WB, IF Abcam Cat#ab195352
m6A, Rabbit, M6A -seq Synaptic systems Cat#202003
H2AK119ub, Rabbit, ChIP Cell Signalling Cat#8240S
H3K27me3, Rabbit, ChIP Diagenode Cat# C15410069
H3K27me3, Mouse, IF Active Motif Cat# 61017
RBM15, Rabbit, WB Proteintech Cat# 10587-1-AP
PCGF3+PCGF5, WB Abcam Cat#ab201510
Alexa 568 anti-mouse IgG, Goat, IF Life Technologies cat#A11031
Alexa 488 anti-mouse IgG , IF Life Technologies cat#A11029
Alexa 568 anti-rabbit IgG, Goat, IF Life Technologies cat#A11034
Alexa 488 anti-rabbit IgG, Goat, IF Life Technologies cat#A11008
Anti-rabbit Ig, HPR, Donkey, WB Amersham cat#NA934V
Anti-mouse IgG, HRP, Sheep, WB Amersham cat#NXA931

Validation

In general, we relied on data provided by the manufacturers for validation as well as references in publications

# Eukaryotic cell lines

Policy information about cell lines

Cell line source(s)

mESC: P6D7A5.8 (iXist-Chr17), XY
mESC: iXist-Chr3, XY
mESC: iXist-Chr3 LBR KO, XY
mESC: iXist-Chr3 METTL3 KO, XY
mESC: iXist-Chr3 PCGF3-AID PCGF5 KO, XY
mESC: iXist-Chr3 RBM15 KO, XY
mESC: iXist-Chr3 SPEN KO, XY
mESC: iXist-Chr3 WTAP KO, XY
mESC: iXist-ChrX, XX
mESC: iXist-ChrX LBR KO, XX
mESC: iXist-ChrX RBM15 KO, XX
mESC: iXist-ChrX SPEN KO, XX
mESC: iXist-ChrX WTAP KO B2.4, XX
mESC: iXist-ChrX WTAP KO B2.6, XX
mESC: iXist-ChrX Xist_DeltaA, XX
mESC: iXist-ChrX Xist_Delta_LBS, XX
mESC: iXist-ChrX Xist_Delta_m6A/3A, XX
mESC: iXist-ChrX Xist_Delta_m6A/11G.7, XX
mESC: iXist-ChrX Xist_Delta_PID, XX

Authentication

Cell lines were validated by high throughput sequencing, Western and/or Southern blot hybridisation, RNA FISH, immunofluorescence as indicated in Supplementary Table 1

Mycoplasma contamination

Cells were confirmed to be free of mycoplasma

Commonly misidentified lines
(See ICLAC register)

No commonly misidentified cell lines were used

# ChIP-seq

## Data deposition

☒ Confirm that both raw and final processed data have been deposited in a public database such as GEO.

☒ Confirm that you have deposited or provided access to graph files (e.g. BED files) for the called peaks.

| | |
|---|---|
| Data access links<br>*May remain private before publication.* | https://www.ncbi.nlm.nih.gov/geo/query/acc.cgi?acc=GSE119599 review token: avqdskuuzjqbzkz |
| Files in database submission | GSM3378085 WT(iXist-Chr3)_NoDox_K27me3_Rep1<br>GSM3378087 WT(iXist-Chr3)_NoDox_K27me3_Rep2<br>GSM3378088 WT(iXist-Chr3)_Dox_K27me3_Rep1<br>GSM3378089 WT(iXist-Chr3)_Dox_K27me3_Rep2<br>GSM3378090 B7(iXist-Chr3_TIR_Pcgf3/5DKO)_NoDox_K27me3_Rep1<br>GSM3378091 B7(iXist-Chr3_TIR_Pcgf3/5DKO)_NoDox_K27me3_Rep2<br>GSM3378092 B7(iXist-Chr3_TIR_Pcgf3/5DKO)_Dox_K27me3_Rep1<br>GSM3378094 B7(iXist-Chr3_TIR_Pcgf3/5DKO)_Dox_K27me3_Rep2<br>GSM3378096 Suz12KO(iXist-Chr3)_NoDox_K27me3_Rep1<br>GSM3378098 Suz12KO(iXist-Chr3)_NoDox_K27me3_Rep2<br>GSM3378100 Suz12KO(iXist-Chr3)_Dox_K27me3_Rep1<br>GSM3378102 Suz12KO(iXist-Chr3)_Dox_K27me3_Rep2<br>GSM3378104 WT(iXist-Chr3)_NoDox_Input_K27me3_Rep1<br>GSM3378106 WT(iXist-Chr3)_NoDox_Input_K27me3_Rep2<br>GSM3378108 WT(iXist-Chr3)_Dox_Input_K27me3_Rep1<br>GSM3378110 WT(iXist-Chr3)_Dox_Input_K27me3_Rep2<br>GSM3378113 B7(iXist-Chr3_Pcgf3/5DKO)_NoDox_Input_K27me3_Rep1<br>GSM3378114 B7(iXist-Chr3_Pcgf3/5DKO)_NoDox_Input_K27me3_Rep2<br>GSM3378116 B7(iXist-Chr3_Pcgf3/5DKO)_Dox_Input_K27me3_Rep1<br>GSM3378117 B7(iXist-Chr3_Pcgf3/5DKO)_Dox_Input_K27me3_Rep2<br>GSM3378119 Suz12KO(iXist-Chr3)_NoDox_Input_K27me3_Rep1<br>GSM3378122 Suz12KO(iXist-Chr3)_NoDox_Input_K27me3_Rep2<br>GSM3378123 Suz12KO(iXist-Chr3)_Dox_Input_K27me3_Rep1<br>GSM3378125 Suz12KO(iXist-Chr3)_Dox_Input_K27me3_Rep2<br>GSM3378127 WT(iXist-Chr3)_NoDox_H2AK119ub_Rep1<br>GSM3378129 WT(iXist-Chr3)_Dox_H2AK119ub_Rep1<br>GSM3378131 B7(iXist-Chr3_TIR_Pcgf3/5DKO)_NoDox_H2AK119ub_Rep1<br>GSM3378133 B7(iXist-Chr3_TIR_Pcgf3/5DKO)_Dox_H2AK119ub_Rep1<br>GSM3378135 Suz12KO(iXist-Chr3)_NoDox_H2AK119ub_Rep1<br>GSM3378137 Suz12KO(iXist-Chr3)_Dox_H2AK119ub_Rep1<br>GSM3378139 WT(iXist-ChrX)_NoDox_K27me3_Rep1<br>GSM3378142 WT(iXist-ChrX)_NoDox_K27me3_Rep2<br>GSM3378144 WT(iXist-ChrX)_Dox_K27me3_Rep1<br>GSM3378145 WT(iXist-ChrX)_Dox_K27me3_Rep2<br>GSM3378147 XistDelPID_NoDox_K27me3_Rep1<br>GSM3378149 XistDelPID_NoDox_K27me3_Rep2<br>GSM3378151 XistDelPID_Dox_K27me3_Rep1<br>GSM3378153 XistDelPID_Dox_K27me3_Rep2<br>GSM3378155 WT(iXist-ChrX)_NoDox_H2AK119ub_Rep1<br>GSM3378157 WT(iXist-ChrX)_NoDox_H2AK119ub_Rep2<br>GSM3378159 WT(iXist-ChrX)_Dox_H2AK119ub_Rep1<br>GSM3378161 WT(iXist-ChrX)_Dox_H2AK119ub_Rep2<br>GSM3378163 XistDelPID_NoDox_H2AK119ub_Rep1<br>GSM3378165 XistDelPID_NoDox_H2AK119ub_Rep2<br>GSM3378168 XistDelPID_Dox_H2AK119ub_Rep1<br>GSM3378170 XistDelPID_Dox_H2AK119ub_Rep2<br>GSM3378172 WT(iXist-ChrX)_NoDox_Input_Rep1<br>GSM3378174 WT(iXist-ChrX)_NoDox_Input_Rep2<br>GSM3378176 WT(iXist-ChrX)_Dox_Input_Rep1<br>GSM3378178 WT(iXist-ChrX)_Dox_Input_Rep2<br>GSM3378180 XistDelPID_NoDox_Input_Rep1<br>GSM3378182 XistDelPID_NoDox_Input_Rep2<br>GSM3378184 XistDelPID_Dox_Input_Rep1<br>GSM3378186 XistDelPID_Dox_Input_Rep2 |
| Genome browser session<br>(e.g. UCSC) | The bigwig files deposited in GEO database can be directly loaded into IGV or UCSC for visualization |

## Methodology

| | |
|---|---|
| Replicates | See above files name with replicate number |
| Sequencing depth | paried-end sequencing (2 x 80) at 35-60 million reads per sample |
| Antibodies | H3K27me3 (Diagenode C15410069, rabbit polyclonal) H2AK119ub (CST #8240S, rabbit mAb D27C4) |
| Peak calling parameters | not relevant |
| Data quality | fastqc was employed to examine the fastq quality |
| Software | STAR (v2.4.2a) |

Software

Bowtie2 (v2.3.2)
featureCount (v1.5.2)
SNPSplit (v0.2.0)
Bedtools (v2.17.0)
Samtools (1.3)
DANPOS (2.2.2)
HiC-Pro (v2.8.0)
ChromHMM (v1.11)
